# Vertebral body tethering: An alternative to posterior spinal fusion in idiopathic scoliosis?

**DOI:** 10.3389/fped.2023.1133049

**Published:** 2023-03-14

**Authors:** Ahmad M. Hammad, Massimo Balsano, Alaaeldin A. Ahmad

**Affiliations:** ^1^ Department of Orthopedics Surgery, American University of Beirut, Beirut, Lebanon; ^2^ Regional Spinal Department, University and Hospital Trust, Verona, Italy; ^3^ Department of Pediatric Orthopedic Surgery, Palestine Polytechnic University PPU, Hebron, Palestine

**Keywords:** pediatric scoliosis and kyphosis, vertebral body tethering, posterior spinal fusion, idiopathic scoliosis, tether breakage, overcorrection

## Abstract

**Introduction:**

Skeletally immature patient with adolescent idiopathic scoliosis (AIS) whose curves continue to progress despite bracing should be treated surgically. Vertebral body tethering (VBT) is a non-fusion, compression-based, growth preserving alternative to posterior spinal fusion (PSF) based on the concept of ‘growth modulation’ to prevent possible functional complications secondary to fusion while correcting scoliotic deformity. This review aims to shed light on the indications of VBT, short- and medium-term outcomes, describe the surgical technique and associated complications, and to compare its efficacy to that of PSF.

**Methods:**

A review of peer-reviewed literature on VBT as a surgical technique, its indications, outcomes, complications, and comparison with other surgical interventions to correct AIS was conducted in December 2022.

**Results:**

Indications remain controversial and mainly include stage of skeletal maturity based on radiographic markers, curve location, magnitude and flexibility, and presence of secondary curve. Assessment of VBT clinical success should not be restricted to improvement in radiographic parameters but should include functional results and patient-centered outcomes, improved body image and pain, and durability of outcomes. In contrast to fusion, VBT seems to be associated with preserved spinal growth, shorter recovery, potentially better functional outcomes, less motion loss but possibly less curve correction.

**Discussion:**

Yet still, with VBT there exists a risk of overcorrection, construct breakage or failure of procedure which require revision and at times conversion to PSF. Patient and family preferences must be accounted for acknowledging gaps in knowledge, attributes and drawbacks of each intervention.

## Introduction

Scoliosis is a deviation of the lateral curvature of the spine beyond 10°. It is a three-dimensional spinal deformation with an incidence rate of 3% worldwide (1, 2). Adolescent idiopathic scoliosis (AIS) is the most common form of scoliosis among pediatric population between 10 and 18 years of age. By definition, AIS implies an etiology that is unknown and not related to a congenital, syndromic or neuromuscular condition (3).

Multiple interventions were devised to correct the scoliotic deformity, halt the progress of the disease, and optimize the quality of life of AIS patients. The decision on treatment plan varies based on curve severity and patient/family preference. The treatment ranges from no intervention to non-surgical management including physical therapy and bracing, and finally surgical intervention.

Posterior spinal fusion (PSF) with pedicle screw instrumentation is indicated in patients with advanced Cobb's angle who failed conservative treatment with evidence of progression of clinical imbalance and radiographic worsening, and have been the traditional method for scoliosis correction. Recently, however, there has been concerns about the effect of spinal fusion on the spine in terms of biomechanics and flexibility (4). As such, and in line with the concept of growth modulation, vertebral body tethering (VBT) has been introduced as a new therapeutic modality to overcome the functional complications associated with PSF by providing a means for children to harness remaining spinal growth to produce correction (5, 6). Initially, and based on biomechanical findings established on animal studies, this technique was found to modify the curvature while preserving spinal motion (7). The ultimate goal of tethering is to create a more normal spinal contour while preserving functional motion (5).

AIS is disproportionate in nature across all planes resulting in lateral deviation and scoliotic deformity, hypokyphosis (anterior-posterior column length mismatch), associated with rib and lumbar prominence and vertebral bodies rotation (5, 6). As such, decreasing both anterior and lateral growth through applying compression on the convex and anterior sides of the vertebrae while promoting and allowing growth on the concave and posterior aspects would result in spinal realignment based on Hueter-Volkmann principle (5). The success of VBT is assessed clinically through radiographic findings and durability of outcomes associated with pain reduction and improvement of body image (8).

This paper aims to provide an update on VBT and shed light on the short- and medium-term outcomes following VBT in AIS patients, the surgical technique and associated complications, and to compare its efficacy to that of PSF.

## Materials and methods

### Search strategy

A review of peer-reviewed literature on VBT as a surgical technique, its indications, outcomes, complications, and comparison with other surgical interventions to correct AIS was conducted in December 2022. The databases reviews were Medline and Pubmed. Keywords used across searches were variants of the following: vertebral body tethering, posterior spinal fusion, idiopathic scoliosis, tether breakage, overcorrection, indications, surgery and comparison.

### Eligibility criteria

Peer-Reviewed journal articles that involve description of VBT surgical technique, indications, outcomes, complications, and comparison with PSF among AIS patients were included. Excluded from this review were articles not related to VBT as a scoliotic correction alternative for AIS, few articles that were part of a systematic review and/or metaanalysis and few articles related to surgical technique or device, anesthesia delivery and pulmonary complications that were beyond the scope of our study aim.

### Procedure

The authors of this study independently reviewed both selected databases for all titles and abstracts and resolved any difference regarding full-text inclusion *via* consensus. The authors then abstracted data across all included studies independently concerning surgical technique, indications, outcomes, complications, and comparison of VBT with PSF to correct AIS. Findings then were compared, and any discrepancies were resolved amongst the authors through active discussions.

### Ethics

Institutional review board approval was not required for this review.

### Indications of vertebral body tethering

VBT has been applied off-label for around 10 years before it was approved in 2009 by FDA as a humanitarian device exemption (HDE) allowing its widespread use. This created a paradigm shift for AIS correction requiring rigorous evaluation of this novel available technique (9).

VBT is a non-fusion surgical technique for correction of scoliosis in skeletally immature children (9). Current FDA indications include a skeletally immature individual age 8–16 with major Cobb angle 35°–60° involving thoracic, lumbar or thoracolumbar curves that failed or did not tolerate bracing (4, 9). Skeletal maturity is defined as Sanders bone age ≤5 or Risser grade ≤2, and curves ≥65° are thought to be too severe for tethering.

VBT is proposed for idiopathic curves only since syndromic curves are unpredictable and may require more reintervention. Lumbar curves are not absolute contraindication for VBT which has been described for thoracic curves, hence, particular caution is needed when performing lumbar correction through open or mini-laparotomy for safe and easy access (10). Newton et al. suggested that tethering more than 1 curve makes correction unpredictable (5). Similarly, left sided thoracic curves are not an absolute contraindication but the surgeon must be aware of an associated syndromic condition and that a left-sided surgical approach carries a higher vascular risk and is thus high demanding (10). Thoracic kyphosis >40° is another relative contraindication whereas a curve with 50% flexibility (40°–60° on supine bending radiograph, bending ≤30°) and rib hump <20° is an ideal candidate for VBT (6, 11).

A study by Krakow et al. on all AIS patients treated with PSF or VBT was conducted to determine the proportion of patients who could have been tethered prior to approval. It was found that 75/359 patients (20.9%) of patients met FDA criteria of VBT and should have undergone tethering when in fact only 18/75 (25%) underwent VBT and the rest were treated with PSF. The study found that an ideal tethering candidate is a Sanders ≤3 at the time of operation (4).

The interplay between skeletal maturity and curve magnitude in relation to success of surgical treatment is a particularly poignant one in VBT. Takahashi et al. identified that curve correction rate significantly varies and is almost double in Sanders 2 compared to Sanders 3 children (12). Another study conducted by Shaw et al. to assess surgeon variability in management of AIS recommended that patients with Sanders ≤3 and smaller curve magnitude were more likely to be advised for VBT but neither age nor flexibility of the spine influenced the decision of choosing between PSF or VBT (13).

When a secondary curve is present VBT might become less attractive (5). Hoernschemeyer et al. identified 5 different subgroups of patients who would benefit from different tethering modalities: single thoracic curve treated with thoracic tether only, both thoracic and lumbar curve treated with thoracic tether and lumbar brace, left thoracolumbar curve treated with thoracic and lumbar tether, large main thoracic and lumbar curves treated with thoracic and lumbar tether, and long thoracic curve treated with single tether (14). Shaw et al. found out that surgeons with high VBT volume ≥11 cases/year were more likely to recommend VBT as compared to surgeons with lower volumes and the former surgeons had higher recommendation rate of VBT to Lenke 1A/3/5/6 curves. Additionally, high VBT volume surgeons trended towards instrumenting more vertebral levels than less VBT volume surgeons (13).

Although VBT has emerged as a viable surgical option for management of AIS, it is still in the process of obtaining full approval by FDA. The candidates for VBT include AIS patients with fused triradiate cartilage, Sanders ≤5, and ≥10 years of age, i.e., AIS with potential spine growth. However, with technique still flourishing with time, the indications to identify the suitable patient that would benefit the most from VBT are yet to be clearly defined and are likely to shift or modify over time. Moreover, a thorough discussion with the patient and family is of paramount importance to elucidate their goals and explain the risk/benefit of each procedure in terms of maximum correction, potential growth, and flexibility prior to choosing the surgical modality.

### Surgical technique and considerations

Appropriate timing of VBT is of paramount importance; early VBT is at risk of overcorrection and late VBT may not correct curve optimally with risk of tether breakage (6). Any patient planned to undergo vertebral body tethering should be optimized preoperatively.

The procedure is carried out with the patient placed in strict lateral decubitus position and using single lung ventilation (right lung deflated intraoperatively) with the convex side of deformity facing upwards (10, 15). Instrumentation is carried from end-to-end vertebrae and preoperative screw trajectory planning is necessary under fluoroscopy to plan portal placement. The spine is accessed *via* an open or mini-open thoracotomy or thoracoscopic approach *via* 1–2 posterior axillary line portals for screw placement and 1 anterior axillary line portal for scope placement. Parietal pleura is opened over the spine and segmental vessels are then ligated or mobilized, followed by vertebral body exposure (10). Under fluoroscopy guidance, staples are placed anterior to rib head followed by a single bicortical screw placement in each instrumented vertebra in single tethering compared to dual screw placement and double cords in double tethers which is reserved for lumbar tethers (14, 16, 17). Afterwards, a polyethylene tether can be placed in a cephalad to caudal direction and under fluoroscopy a tensioner is used to apply sequential compression across each level starting cranially to bring the tilted discs into neutral alignment (16). Once screws are confirmed to be parallel, then set screws are locked into position. Finally, the thorax is irrigated, lung reinflated, and a chest drain is inserted (6, 10).

Thoracic tethers are typically performed thoracoscopically whereas thoracolumbar/lumbar tethers require a mini-open approach. A rib sparing thoracotomy with incision of diaphragm posteriorly allow access to T11–L1 vertebrae through a plane anterior to the psoas. For lower lumbar levels, a second incision is required *via* an anterolateral retroperitoneal approach (16). In the mini-open technique, segmental vessels can be mobilized and it facilitates spinal derotation and cord tightening with more ease. A thoracoscopic approach, however, necessitates segmental vessel ligation and is associated with decreased pain postoperatively, better pulmonary function and better cosmesis. Yet still, no study is available comparing such approaches (6).

The aim is to restore residual on-the-table curvature to 15°–20° in immature patients and to maximal correction in mature patients. A combination of manual force, segmental compression and tensioning, apical translation and derotation is needed for maximal correction. Surgeons must take care to tighten the cable sequentially rather than globally (15) with maximal tightness at the curve apex while minimizing it at the upper (UIV) and lower (LIV) instrumented vertebrae which are yet to be established (10, 13). The amount of tightness is guided intraoperatively by fluoroscopy. Finite models showed that anterior tethering and 200N tightening provide better correction in all planes (6). Patients expected to have less bone remodeling require a more robust construct through using double cords with 2 rows of screws to withstand forces of daily activities while permitting remodeling (17).

As important as the surgical expertise and technique are for obtaining an optimum output following VBT, several perioperative and intraoperative factors are also required. Intraoperative neuromonitoring should be available to monitor spinal cord function and assess extremities (10). Proper fluoroscopy and C-arm machine are required for conducting the operation. Moreover, basic thoracoscopy skills and surgical skills are required to safely navigate the chest and place the construct, otherwise a thoracic/general surgeon can help and easy the learning curve of the procedure (5).

## Results

### Short-term and medium-term outcomes

#### Correction

Preclinical animal studies were first to show the impact of a flexible tether on growth modulation. On a normal porcine immature model and on a goat model, a tether attached *via* pedicle screws anterolaterally was able to induce and correct spine deformity and change morphology while maintaining disc health and maximizing axial growth (15, 18, 19).

A systematic review by Ratio et al. including 23 studies on 843 patients showed improvement in thoracic curve from 49° to 23° at 2 years with 18% complication rate and 15% reoperation rate, 10% secondary to pulmonary and tether related complication and <5% conversion to PSF (6). The first successful case was reported in 2010 by Crawford and Lenke on an 8-year-old boy with a 40° right thoracic curve treated *via* anterior tethering and noticed to have scoliotic curve correction even to the point of overcorrection (10, 15). The first series was reported by Samdani et al. in 2014 on 11 AIS patients treated with VBT and found after 2 years to have around 70% correction of thoracic and lumbar curves from 44.2° to 13.5° and 25.1° to 7.2°, respectively, with 2 patients needing reoperation to loosen the tether and prevent overcorrection (20). Similar findings were also reported in 2015 by Samadani et al. on 32 patients with thoracic correction from 42.8° to 17.9° with 1 case of atelectasis and 3 cases of overcorrection (21). Boudissa et al. reported 1-year results of 6 patients with correction of thoracic (45°–38°) and lumbar curves (33°–25°) following VBT with no complications and no patients requiring fusion (22).

**Figure 1:**
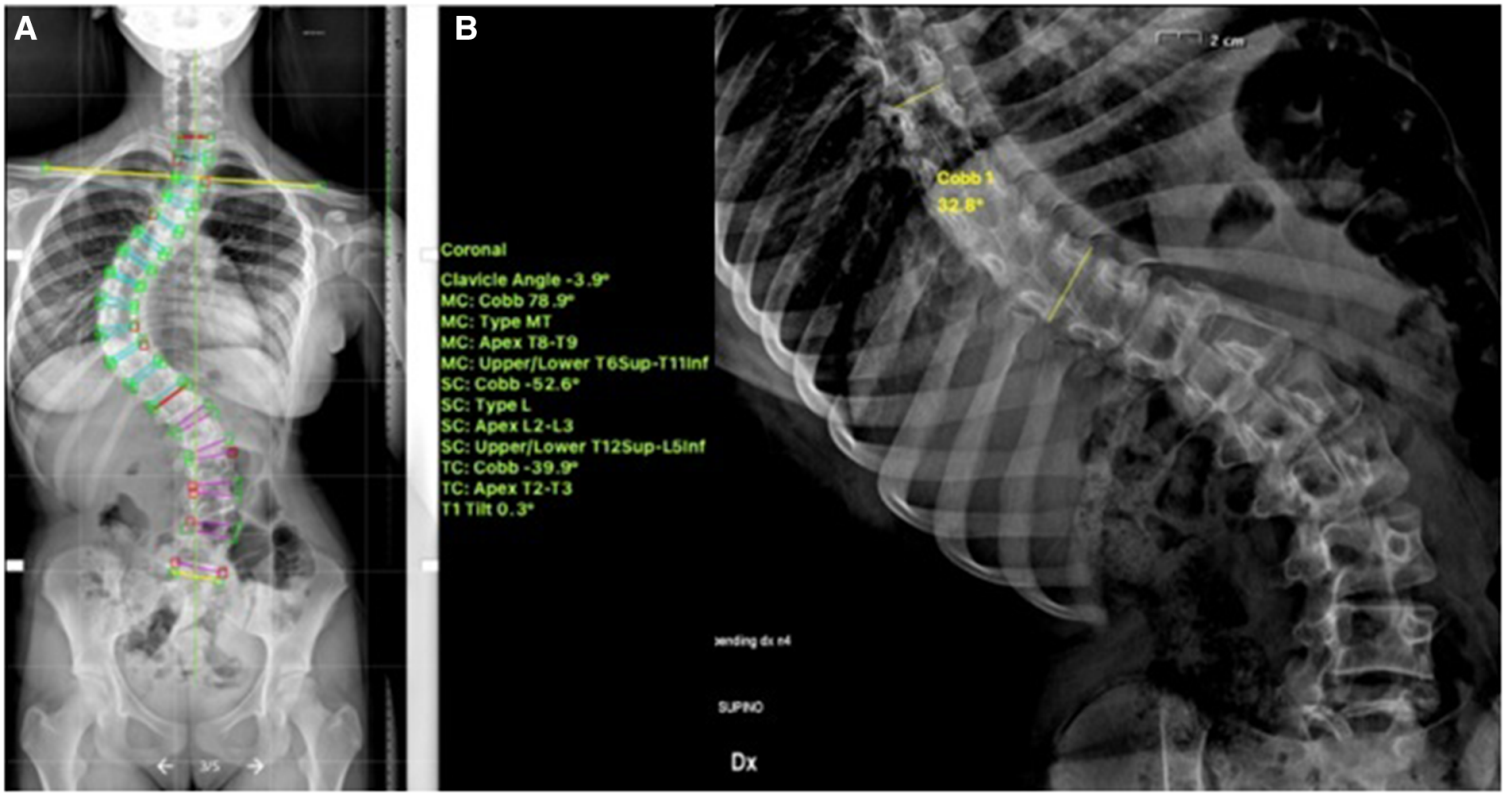
13-years old female (Sanders 5) with (**A**) a major thoracic curve 52° (**B**) corrected to 32° on bending.

**Figure 2:**
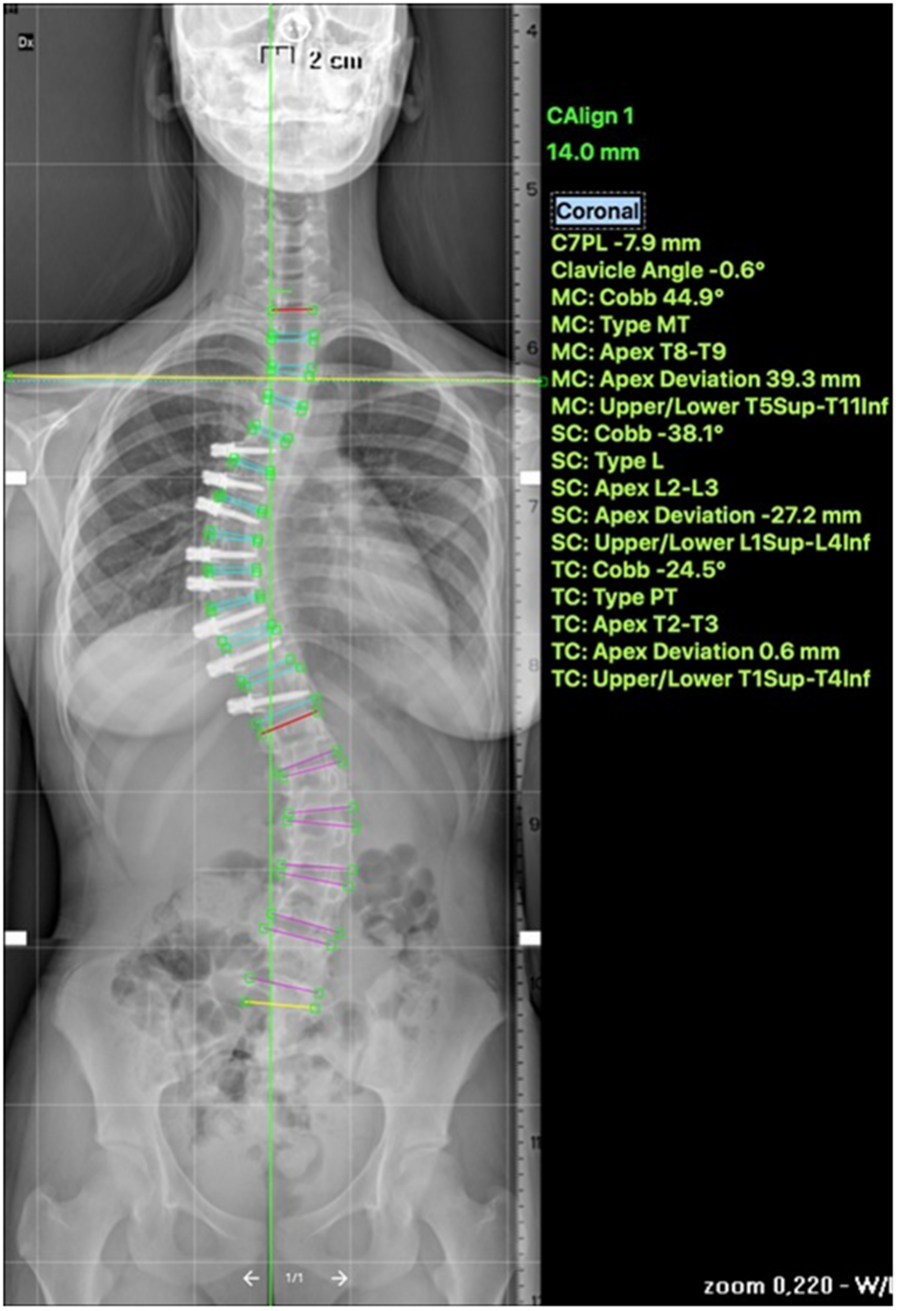
Post operative—anterior tethering T5–12 was done with correction of the thoracic curve to 45°.

**Figure 3:**
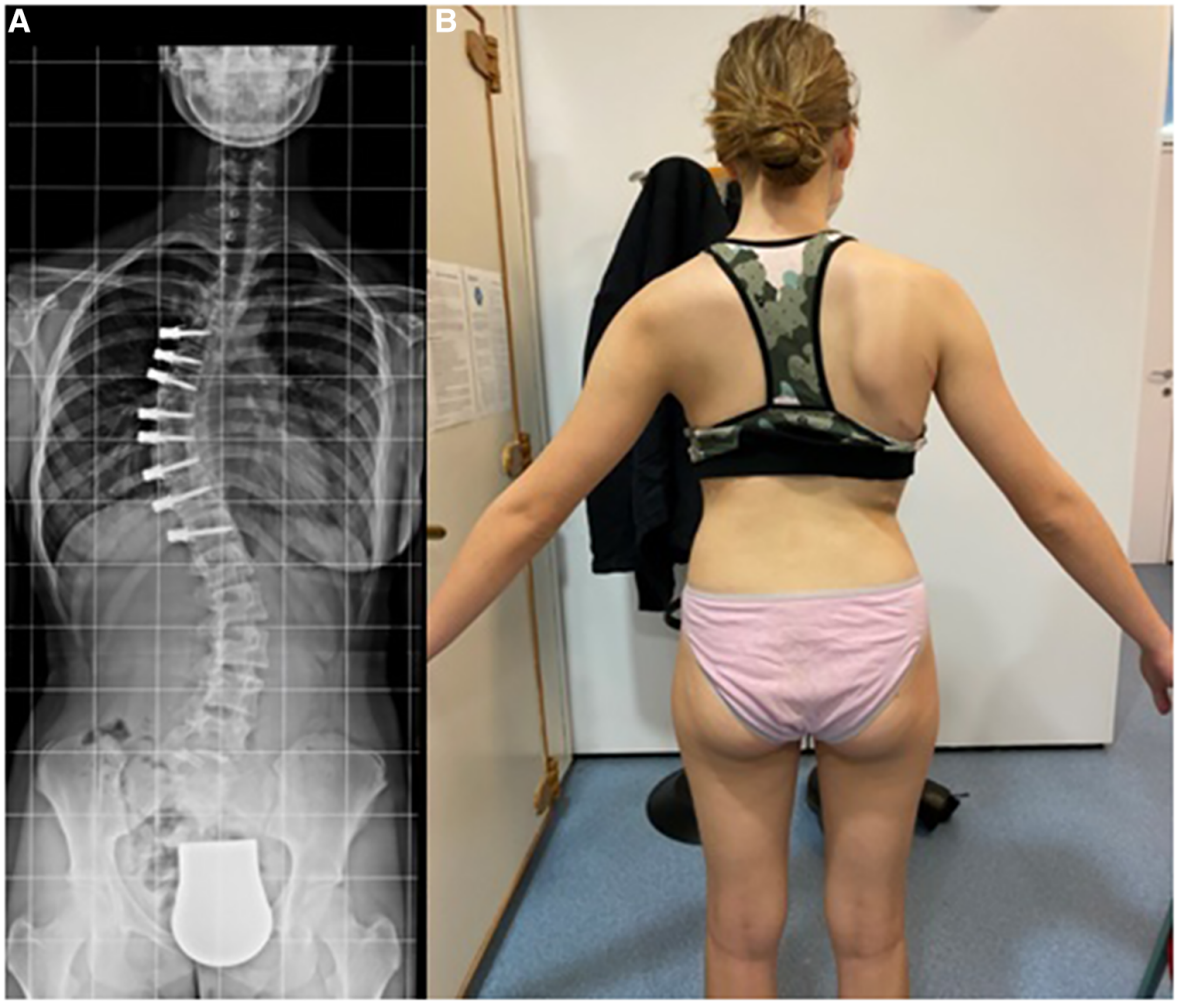
1-year post-tethering, patient is well compensated; (**A**) radiographic compensation, (**B**) apparent compensation.

Recently, multiple studies have reported on the outcomes >2 years post tethering. Newton et al. reported in 2018 on 2–4 years follow-up date on 17 skeletally immature (Risser 0) patients with 59% clinical success and improvement in thoracic Cobb's angle from 52° to 27° and 7 patients (41%) requiring reoperation to prevent overcorrection, address lumbar curve progression, revise broken tether, or undergo revision and posterior spinal fusion (23). Wong et al. reported on 4-year follow-up in cohort of 5 patients between 9 and 12 years of age and found that curve modulation was noted in patients with open triradiate cartilage compared to curve stabilization in those with closed triradiate cartilage (24). Hoernschemeyer et al. reported in 2020 on 29 patients, mean age 12.7 years, treated with VBT and followed up for 3.2 years, and found that tether breakage was suspected in 48% and the revision rate was 21%; only 2 patients at the latest follow-up required fusion and 93% avoided PSF (14). Rushton et al. published on 112 skeletally immature patients with correction of Cobb angle (50.8°–25.7°) and rib hump (14.1°–8.8°); the study showed 71% clinical success (Cobb's angle <35°) at final FU at 37 months and 22% complications with 13% requiring revision (16). The majority of studies on VBT analyzed thoracic curves, Trobisch et al. studied lumbar VBT and reported at 1-year follow-up 66% surgical success with single and double tethering and stable lumbar lordosis (25). Figures 1–3 showcase a 13-year-old female (Sanders 5) that underwent T5-T12 anterior tethering with correction of the curve from 52° to 45° postoperatively and the patient being well compensated at 1-year post surgery.

**Figure 4:**
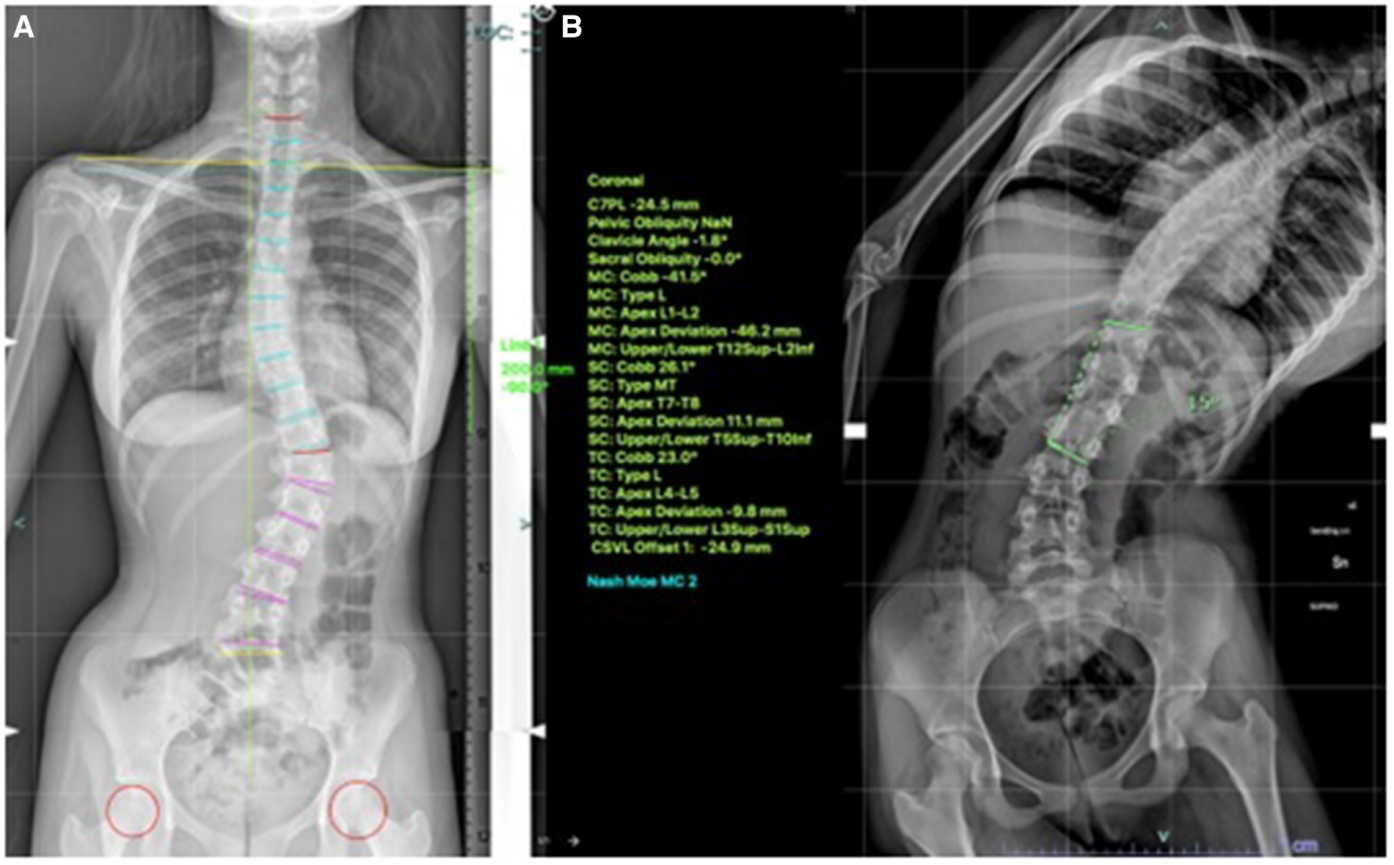
14-years old female (Sanders 7) with (**A**) lumbar curve 42° (**B**) corrected to 15° on bending.

**Figure 5:**
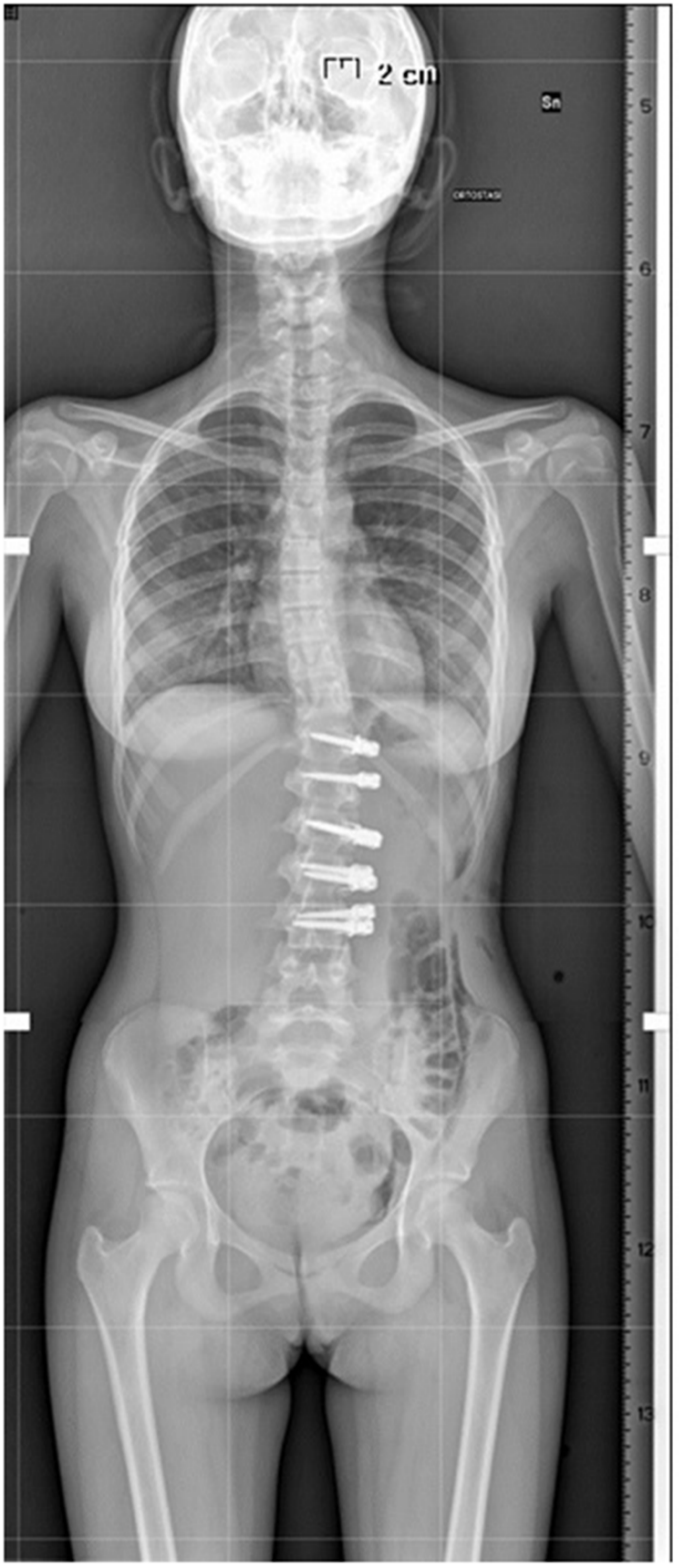
Post operative—anterior tethering T12–L4 with correction of the lumbar curve to 17°.

**Figure 6:**
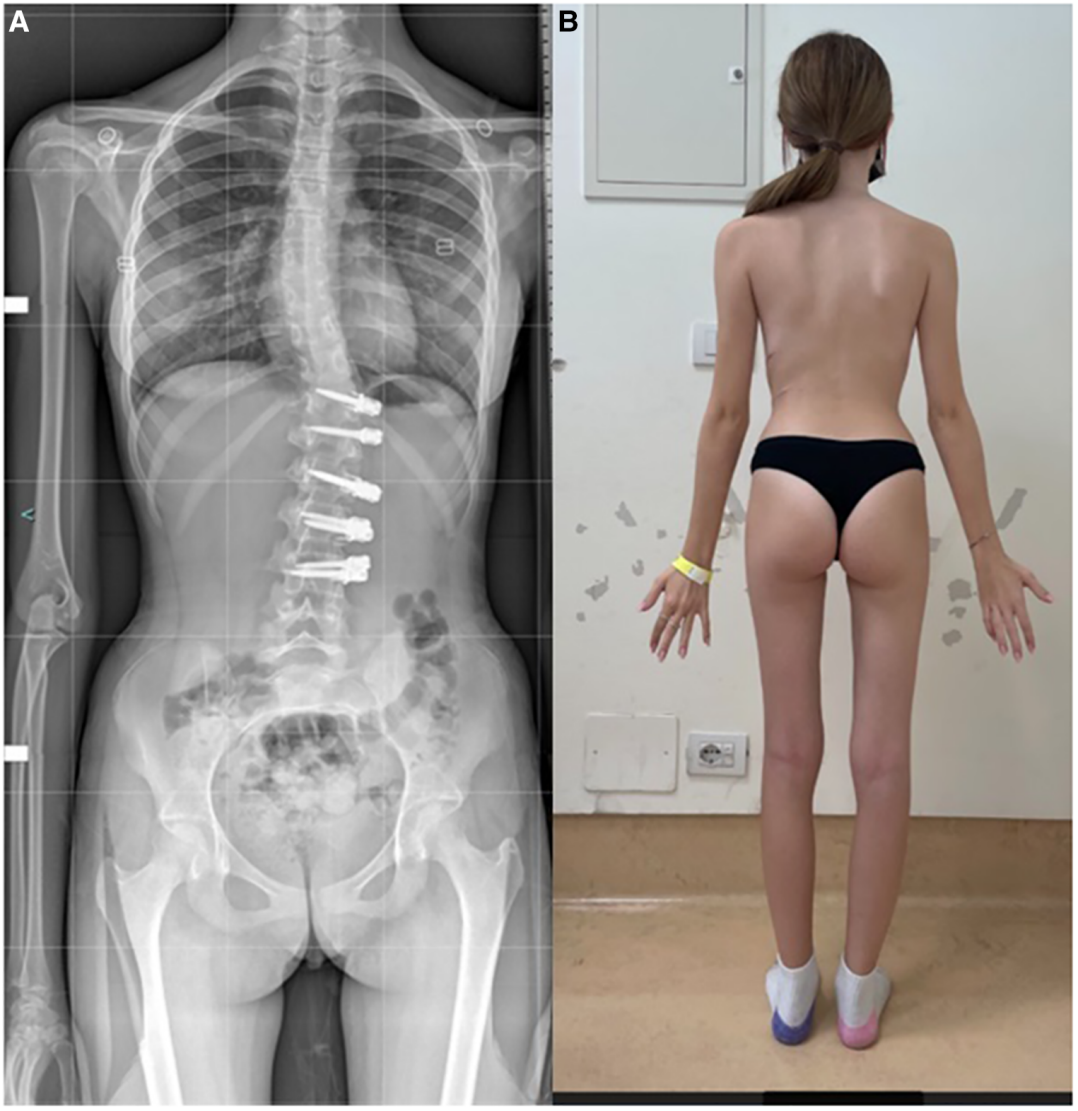
6-months post-tethering; (**A**) radiographically, Cobb angle maintained at 17°, (**B**) patient is apparently well balanced.

VBT for skeletally mature patients continues to be a topic of debate with growing evidence. In patients approaching skeletal maturity, VBT been recently studied as an alternative to PSF that was previously seen as the sole corrective option available (17). Hegde et al. reported on 10 female skeletally mature patients (Risser 4 and Sanders 7) after minimum 1-year follow-up and reported improvement in Cobb's angle from 52° to 15.9°. In skeletally mature patients, VBT is thought to improve the deformity when maximal intraoperative correction is done because growth potential is limited and thus less predictable growth-guided modulation (8, 15). Miyanji et al. published on 55 immature patients Risser 0.5 showing clinical success in 77% and 8% insufficient correction requiring conversion to PSF (19). Alanay et al. reported on mature cohort of Sanders score 6–7 with 100% success, 55% correction and residual curve (9°–27°) at 20 months and Meyers et al. reported on 49 mature patients with 76% success, 41% tether breakage and 2% revision with PSF at 32.5 months (8, 26). Figures 4–6 showcase a 14-year-old female (Sanders 7) that underwent T12-L4 anterior tethering with correction of the curve from 42° to 17° postoperatively and the patient being well balanced at 6-months post surgery.

Rib hump correction is another important key aspect for parents and adolescents considering surgical intervention, whereby resolution of rib hump is correlated with improved outcomes following PSF. Previous studies reported improvement in rib hump ranging between 30% and 45% which adds to the reasons VBT trended as an alternative to PSF (16, 20, 21, 23). Miyanji et al. also reported on improvement on rib hump in addition to lumbar prominence improvement from 3.5° to 2.3° (19).

Such results are promising for both immature and mature patients with remaining spinal growth who would benefit from VBT as an alternative method for curve correction while preserving growth. Yet still, there exists a risk of overcorrection, construct breakage or failure of procedure which require revision and at times conversion to PSF. Successful VBT operation are seen with smaller preoperative major curve and curves that are more flexible (8, 16) and neither age nor Risser staging contributed to success or failure of the procedure (8).

#### Radiographic and global parameters

Just as important as the coronal measurements are in assessing outcomes of VBT, other radiographic parameters including sagittal findings, thoracic kyphosis and lumbar lordosis are also important to highlight. Buyuk et al. highlighted that sagittal spinal motion is preserved after VBT (27). Hoernschemeyer et al. reported >85% neutral sagittal vertical axis post-VBT as compared to 62% pre-VBT along with significant difference in thoracic kyphosis and lumbar lordosis postoperatively (14). Baroncini et al. reported an increase in thoracic kyphosis but no change in lumbar lordosis and decrease in pelvic tilt and sagittal vertical axis at 2 years postoperatively; however, when lumbar curve was instrumented there was no kyphotic effect on lumbar lordosis (28). As such VBT despite not allowing direct sagittal correction, it seems to influence advantageously the sagittal parameters without a kyphotic effect on lumbar lordosis. Trobisch et al. found that the use of double tethering did not have a kyphotic effect on the lumbar lordosis which could be explained by derotation or nucleus reposition during VBT (25). Additionally, decreased pelvic tilt and sagittal vertical axis indicate better global balance, balanced distribution of body mass over hips and improvement in ergonomics of erect position (28).

### Complications

Vertebral body tethering can effectively correct scoliotic deformities, yet this surgical approach does not come without complications and need for further revisions. A tether is not expected to hold forever but to provide stability and control curve progression until skeletal maturity, disc maturity and remodelling. A systematic review by Zhang et al. including 25 studies of 1,052 patients investigated VBT outcomes and reported 21.3% tether breakage, 6.9% pulmonary complications and 4.2% overcorrection with 13.1% requiring revision (29).

#### Tether breakage

Tether breakage (TB) is the most common complication seen in patients undergoing VBT. Most breakages are asymptomatic and do not significantly impact spinal curve thus most not requiring management, whereas some AIS patients are prone to lose correction following TB (30). A tether is radiolucent and not visible on radiograph; hence breakages can only be suspected. Early TB is defined as >5° angulation between 2 adjacent screws on 2 consecutive x-rays within first postoperative year. Trobisch et al., found that the latter definition underestimates incidence of TB; the authors report that only 56% could be diagnosed and that every other TB remains undiagnosed where among 36 real breakages only 20 were suspected and 16 were thought to be intact of which 2 underwent revision for scoliosis progression (31).

Baroncini et al. reported in 2022 high incidence of early TB 55% (58/105), more in lumbar curves as compared to thoracic (71% vs. 29%) with large curvature, limited flexibility and a higher residual postoperative curve being risk factors for early breakage; age and skeletal maturity were not found to increase TB risk (30). 6 of these patients lost correction and 2 had scoliosis progression requiring revision surgery. Baroncini et al. then investigated the influence of timing of TB on clinical results and found that patients with no TB or TB >12 months had similar improvement in Cobb's angle but better than that seen in patients with early TB within 6 and 12 months (4.8°/7.8° vs. 15.8°/13.8°) (32). Another study by Shankar et al. reported 27% (18/69) TB at 2-years follow-up, mostly occurring in major compared to minor curves and in thoracolumbar compared to thoracic tethers (33). It also showed that double cords may not be protective against TB as no difference in breakage was seen between single and double tethering (32% vs. 30%) and there was also no difference in SRS-22 functional scores between patients with intact and broken tethers (33). Newton et al. reported 42%–48% TB incidence rate at 2–5 years post tethering (23, 34). Trobisch et al. in their study on outcomes of lumbar curve correction following single or double tethering noticed 73% asymptomatic breakage rate with limited loss of correction (12.1°) and 90% of patients still having successful or acceptable results at 1 year; 3 patients (10%), all of whom been single tethered, required revision and fusion for loss of correction (25).

Large, rigid curves along with higher range of motion of lumbar curve place the construct under high mechanical load increasing tether wearing and breakage (25, 30, 33). Another explanation for early breakage is attributed to incomplete remodeling process of bony and soft tissues to new shape of the spine as compared to late breakage where most remodeling has already taken place (25). Other potential risk factors are related to implant characteristics (screw shape, tether strength), surgical technique specifics (vertebral level, tether tightening and manipulation), surgeon expertise and learning curve, and patient compliance with postoperative activity restrictions (31).

The aforementioned findings support the fact that a tether plays a fundamental role during the first year and once remodeling process terminates the spine holds its new shape and tether functions is lost (30, 32). Even though, TB is associated with decreased curve correction, it is not associated with clinical success of the procedure or with reduction in quality-of-life post-surgery, nor is it an indication for reoperation (30, 32). Surgical revision is indicated in case of significant loss of correction resulting in dissatisfied patient with a curve >40° and include release of tether, extension or replacement with a new tether or switching to PSF (29, 31). It is noteworthy that compared with previous studies, and after 2020, revision rate decreased from 17.5% to 11% which might be due to better understanding of the indication, surgical technique and optimal intervention time along with development of new constructs to decrease associated mechanical failure.

#### Coronal overcorrection

This is another complication that may arise following VBT and is not completely understood (9). It tends to occur in immature patients with Sanders score ≤2 or with open triradiate cartilage, i.e., have more growth remaining. Newton et al. was first to report that skeletally immature patients continue to correct and even go to overcorrection to 121% as compared to mature adolescents whose tethers act as permanent tether rather than as true growth modulators (9, 23, 24). Alanay and colleagues noticed that overcorrection and mechanical complications are more common in Sanders 2 group (26). Such observation is avoided by delaying VBT until patient skeletally matures to Sanders stage 3 and is treated with removal of tether to avoid further curvature modification (23, 26). Lateral decubitus position and screw placement within the vertebrae are also important factors that contribute to curve correction and should be factored in as suggested by Cobetto et al. (35).

#### Other complications

Non-revision related complication following VBT include atelectasis, pulmonary edema, hemothorax/chylothorax, Horner syndrome, wound infection, spinal canal penetrations among others (34, 36). Pulmonary complications are the most frequent non-mechanical complications occurring within 6 weeks of VBT and all resolve without remaining complaints. Trobisch et al. reported 14/140 (10%) pulmonary complications following VBT; pleural effusion seems to be more common in patients with diaphragm split, despite no conclusive evidence yet (37). Additionally, Alanay et al. reported 12% (4/31) pulmonary complications and Wong et al. observed 60% (3/5) pulmonary complications in their study whereas Boudissa et al. did not report any complication in 6 VBT treated patients (22, 24, 26). These complications may be avoided with limited pleural opening, diaphragm suturing in case of a split, pulmonary training, and chest tube management (37).

### Tethering vs. spinal fusion

VBT modulates spinal growth by compressing the anterior spine for treatment of skeletally immature AIS as an alternative to the gold standard treatment PSF. Even though, VBT can successfully reduce the curvature, it is hypothesized to be less consistent and less predictable as compared to PSF.

Newton et al. reported in 2020 a comparative series of AIS patients, Risser ≤1 age 12 and Cobb's angle 40°–67°, treated with VBT and matched to a cohort treated with PSF with minimum 2-years follow-up. The authors documented correction in both groups with VBT group having more residual deformity (33° vs. 16°) and higher number of revisions (9 vs. 0) than PSF. At final follow-up at 3.4 years, 52% of patients in VBT group were considered successful with residual curve <35° compared to all patients in PSF group with no differences in patient reported outcomes between both groups. VBT group grew significantly more with mean height gain 15 cm compared to 9 cm in PSF group (34). Additionally, at 2 years follow-up, Mattew et al. reported that curve correction was superior at all timepoints in immature patients following PSF than after VBT (96% vs. 77%) with 19% complication rate in VBT group (36).

Assessment of clinical success should not be restricted to improvement in radiographic parameters but should include functional results and patient-centered outcomes, improved body image and pain, and durability of outcomes. Several previous studies reported worse global spinal range of motion following PSF including a study by Danielson et al. on 135 AIS patient with 20 years follow-up that showed significant reduction in lateral and anterior bending flexibility, muscle endurance and motor trunk muscle strength with reduced physical functioning and limitation in social activities and daily activities (38, 39). A recent study by Maksimovic et al. comparing global spine range of motion following AIS patients’ curve correction following PSF or VBT showed preservation of spinal motion in the transverse plane following VBT whereas PSF was associated with worse outcomes during thoracic and total axial twist (40).

In the first study of its kind, Pehlivanoglu et al. compared the clinical outcomes of VBT to PSF and detected better lumbar range of motion, lumbar bending flexibility, flexor/extensor trunk endurance and trunk muscle strength and rotation following VBT at 37 months. In terms of functional outcomes, Pehlivanoglu et al. also noticed that VBT resulted in superior life quality scores, total SRS-22 and SF-36, and higher satisfaction as compared to PSF (7). Another study by Qiu et al. showed more thoracic flexibility on bending radiographs in VBT group when compared to PSF cohort but both groups had similar health related quality of life scores (41). Pahys et al. noticed loss of motion across all directions following PSF whereas VBT-patients maintained extension and rotation but had loss of flexion and side-bending after 2 years. SRS-22 scores were similar between both groups at last follow-up (42). Such findings could be explained by the avoidance of posterior paraspinal muscles dissection and applying a minimally invasive thoracoscopic approach and that patients undergoing tethering tend to have more flexible curves prior to surgery and are thus able to maintain it afterwards. VBT is less invasive and thus patients are expected to resume normal daily activities and sports faster than their PSF counterparts (6).

A study by Theologis et al. comparing VBT and PSF at index and revision surgeries showed that VBT procedure had shorter operation time, fewer spinal levels instrumented, less blood loss with similar length of in-hospital stay but higher costs and more revision operations compared to PSF (43). Mathew et al. found shorter recovery (lower operative time, blood loss, length of stay) in patients undergoing VBT group compared to those undergoing PSF (36). Average cost of VBT was greater than PSF at index surgery due to implant cost and postoperative care but similar at readmission/revision surgery in case of VBT related overcorrection/curve progression or PSF related junctional deformity (43). However, a study by Shin et al. comparing VBT to PSF showed that VBT was associated with higher rates of complications (26% vs. 2%) and reoperation (14.1% vs. 0.6%) compares to PSF at 36 months follow-up (44).

Even though deformity correction is more predictable in patient undergoing PSF, when the goal is to preserve truncal motion and avoid fusion, while reducing curvature and risk of scoliosis progression with maturity, then VBT is a non-inferior alternative technique to fusion. It is noteworthy that long-term follow-up studies are indeed needed to truly assess the impact of VBT on spinal range of motion.

## Discussion

As the endeavor to find the best surgical treatment of AIS continues to merit excitement, clinicians should remember that bracing is the gold standard of nonsurgical treatment in AIS patients, and is associated with decreasing the risk of curve progression and few complications (45). VBT has emerged as a viable surgical option for management of AIS that preserves truncal motion and avoids fusion, while reducing curvature and risk of scoliosis progression with maturity. With VBT still flourishing with time, the indications to identify the suitable patient that would benefit the most from this technique are still not clearly defined and are likely to shift or modify over time. Moreover, a thorough discussion with the patient and family is of paramount importance to elucidate their goals and explain the risk/benefit of each procedure in terms of maximum correction, potential growth, flexibility, and complications prior to choosing the surgical modality. PSF continues to be the most widespread surgical option with a well-established complication and reoperation rates. Hence, further studies are indeed needed to assess whether VBT is a superior approach, identify the ideal candidate for VBT, explore the long-term outcomes, and monitor for future complications or need for reintervention following tethering.

To date, there are no randomized controlled trials or prospective studies comparing VBT to PSF in terms of outcomes, hence an evidence-based recommendation of which surgical modality is better is still lacking. All available outcomes are short- and medium-term and still there exists no long-term outcomes. It is true VBT serves the purpose of spinal correction and preserve growth, however and as a surgical intervention, it comes with a range of complications that might interfere with the treatment course and require revision. Additionally, the long term outcomes and complications following VBT are yet to be investigated. A successful VBT is a costly operation; hence when complicated, the patients and their families need to be aware of the cost-effectiveness of PSF compared to VBT. Additionally, the ideal bone age, ideal curve, and optimal timing to tethering continue to be gray zones that need clarification. Furthermore, another important step in this novel procedure is to clarify the surgical indications and unify the general procedural steps including levels of instrumentation and tensioning techniques. Another limitation is the breakage of the construct which necessitates a more durable, fatigue resistant cable to overcome this frequent complication. Currently, several ongoing clinical trials are conducted to show effectiveness and safety of VBT, propose strategies to decrease/avoid complication and failure, and compare it to PSF (46).

## Data Availability

The original contributions presented in the study are included in the article/Supplementary Material, further inquiries can be directed to the corresponding author.
